# Overlapping point cloud registration algorithm based on KNN and the channel attention mechanism

**DOI:** 10.1371/journal.pone.0325261

**Published:** 2025-06-02

**Authors:** Yangzhuo Chen, Fengjiao Guo, Jingang Liu, Siling Dai, Jia Huang, Xiaowen Cai

**Affiliations:** 1 School of Mechanical Engineering and Mechanics, Xiangtan University, Xiangtan, China; 2 School of Automation and Electronic Information, Xiangtan University, Xiangtan, China; 3 Hunan Shaofeng Institute of Applied Mathematics, Xiangtan, China; 4 Xingxiang College of Xiangtan University, Xiangtan, China; University of Lagos Faculty of Engineering, NIGERIA

## Abstract

With the advancement of sensor technologies such as LiDAR and depth cameras, the significance of three-dimensional point cloud data in autonomous driving and environment sensing continues to increase.Point cloud registration stands as a fundamental task in constructing high-precision environmental models, with particular significance in overlapping regions where the accuracy of feature extraction and matching directly impacts registration quality. Despite advancements in deep learning approaches, existing methods continue to demonstrate limitations in extracting comprehensive features within these overlapping areas. This study introduces an innovative point cloud registration framework that synergistically combines the K-nearest neighbor (KNN) algorithm with a channel attention mechanism (CAM) to significantly enhance feature extraction and matching capabilities in overlapping regions. Additionally, by designing an effectiveness scoring network, the proposed method improves registration accuracy and enhances system robustness in complex scenarios. Comprehensive evaluations on the ModelNet40 dataset reveal that our approach achieves markedly superior performance metrics, demonstrating significantly lower root mean square error (RMSE) and mean absolute error (MAE) compared to established methods including iterative closest point (ICP), Robust & Efficient Point Cloud Registration using PointNet (PointNetLK), Go-ICP, fast global registration (FGR), deep closest point (DCP), self-supervised learning for a partial-to-partial registration (PRNet), and Iterative Distance-Aware Similarity Matrix Convolution (IDAM). This performance advantage is consistently maintained across various challenging conditions, including unseen shapes, novel categories, and noisy environments. Furthermore, additional experiments on the Stanford dataset validate the applicability and robustness of the proposed method for high-precision 3D shape registration tasks.

## Introduction

A point cloud consists of a collection of three-dimensional points in space [1]. Point cloud registration is an important task in computer vision that aims to find a rigid transformation that aligns a 3D source point cloud with another 3D target point cloud [2]. Effective point cloud registration can not only improve the accuracy of environment perception, but also enhance the adaptability of the system to dynamic changing scenarios. However, in practical applications, point cloud registration still faces numerous challenges. For example, due to the interference of noise points and nonoverlapping points, sampling overlapping points may lead to the incorrect removal of the correct corresponding points in the target point cloud, thereby significantly reducing registration performance.

Feature-based registration methods usually start by extracting geometric features of the point cloud, such as normal vectors, curvatures, or descriptors (e.g., fast point feature histograms (FPFHs) [3], signature of histograms of orientations (SHOT) [4], etc.), and then by matching these features to align the point cloud. Common algorithms such as the random sample consensus (RANSAC) algorithm [5] utilize the principle of random sample consistency to find matching pairs of points in noisy data. Another method is geometry-based registration methods, such as the iterative closest point (ICP) algorithm [6, 7], which performs registration by minimizing the point-to-point distance between the source and the target point clouds. ICP and its variant [8–11] are the most widely used point cloud registration algorithms, but most of them are computationally expensive and sensitive to initialization, noise and outliers. In recent years, the remarkable achievements of deep learning have attracted many researchers to apply it to point cloud registration [12]. Neural network-based data-driven approaches have proven to be more effective in solving many computer vision tasks, with these deep learning methods demonstrating significant improvements in accuracy and robustness compared with traditional registration algorithms. Aoki *et al*. [13] proposed a robust and efficient point cloud registration algorithm PointNetLK that combines deep learning on point sets (PointNet) [14] and the improved Lucasâ€’Kanade algorithm [15] to improve registration in moderate-scale transformations and noisy conditions. Wang *et al*. [16] proposed a DCP network that uses a dynamic graph CNN (DGCNN) [17] to extract the point cloud features and computes the correlation of the points via an attention mechanism. OMNet [18] employs a masking mechanism to counteract the detrimental effects of the partial overlapping of point clouds. RIEnet [19] introduces an interior point evaluation module to identify reliable correspondences. Predator [20] introduces overlapping attention blocks to facilitate the exchange of information between two point clouds. Wang *et al*. [21] proposed PRNet deep learning network that uses Gumbel-Softmax and a direct gradient estimator to sample the key points of the correspondence. Li *et al*. [2] proposed an IDAM network that fuses the local geometric features and distance features of the point cloud and simultaneously improves the registration accuracy by using the mutual supervised hybrid elimination method.

Although existing deep learning methods achieve good performance in point cloud registration, the feature extraction capability is still insufficient when dealing with overlapping regions. Only scanning overlapping regions can lead to registration advantages because nonoverlapping regions are essentially anomalies for registration [22]. However, current registration algorithms are often limited in extracting these features, which affects the overall registration performance. Therefore, improving the feature extraction method to enhance the feature representation of overlapping regions is the key to improving the registration performance of partially overlapping point cloud registration. On this basis, this work proposes a novel point cloud registration method that combines the K nearest neighbor (KNN) algorithm with the CAM to efficiently extract and enhance features in overlapping point clouds. The main contributions of this paper include the following:

(1) KNN and the CAM are combined to achieve a feature extraction and enhancement network; local features of a point cloud are searched with different neighborhoods via KNN, and the ability to describe local geometric structures is enhanced while the importance of features is dynamically adjusted through the CAM to identify key points with rich features.
(2) Designing a Effectiveness score network reduces the impact of incorrectly removing the corresponding points during key point sampling on the registration performance, while combining local geometric and distance features improves the registration accuracy.

## Design of a point cloud registration framework based on dynamic feature fusion and selection

**Fig 1:**
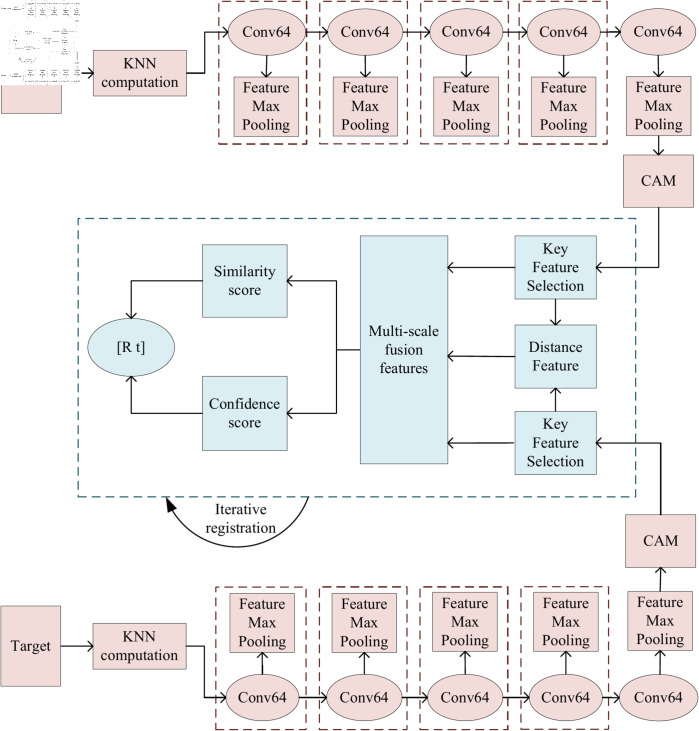
Model architecture diagram.

In this paper, a point cloud registration algorithm based on dynamic feature fusion and selection is proposed. The structure of the algorithm is shown in Fig 1. which consists of four main parts: feature extraction and enhancement, selection of the main features, feature fusion, and generation of the matching probability.

(1) Feature extraction and enhancement phase: The input point cloud data are used to find the proximity points of each point via the KNN algorithm. The neighbor information of the source point cloud is feature-extracted by a convolutional layer, where the convolutional operation captures the local geometric structure of the point cloud and embeds it into a high-dimensional feature space for a more accurate geometric representation. By introducing the CAM, the model can adaptively adjust the feature weights of each point, thus optimizing the feature representation, highlighting the key points, suppressing the redundant information, and further improving the accuracy of the geometric features.
(2) Feature selection phase: To ensure the robustness and accuracy of the model, noise points and outliers are filtered through a specific selection mechanism, and the main features are retained for subsequent processing.
(3) Feature fusion phase: The model combines the retained main features with the relative distance information between points to generate multiscale fused features. Through this fusion, the model can capture geometric information at different scales and utilize the relative distance between points to further enhance the expression of features.
(4) Matching probability generation phase: The model calculates the similarity and effectiveness scores of the features, weights the point cloud according to the scores, and adopts weighted singular value decomposition to solve the final rigid transformation for the accurate registration of the point cloud.

## Design of an overlapping point cloud registration algorithm based on KNN and the channel attention mechanism

### Feature extraction and enhancement

**Fig 2:**
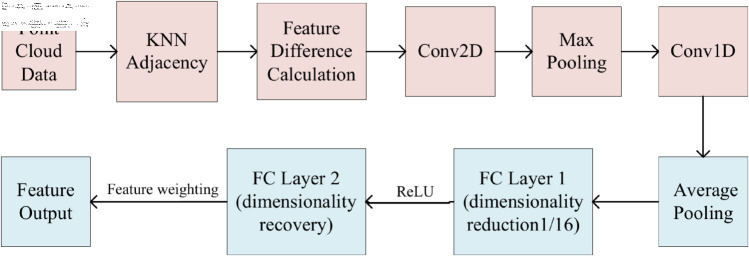
Feature extraction and enhancement module.

The input point cloud data are processed through the adjacency relationships generated by KNN, combined with convolutional layers, to capture local point cloud information at different neighborhood scales [23]. Through multiple information exchanges, the features of each point are updated layer by layer, with the updates being based on the relationships between the point and its neighbors. In the propagation layer, after calculating the feature differences between each point and its neighboring points, these differences are extracted through a 2D convolutional layer. To further compress and aggregate the local features, the maximum feature value is taken from the neighborhood. Finally, the features are further extracted through a 1D convolutional layer. The general process of feature extraction and enhancement module is shown in Fig 2.

These features are then passed to the CAM section. The information from each channel is aggregated through adaptive average pooling, the channel dimension is compressed via a 1D convolutional layer, and a ReLU activation function is applied to introduce nonlinearity. After this, the channel number is restored to the original dimension through a second 1D convolutional layer, and channel attention weights are generated via a sigmoid activation function to indicate the importance of each channel. Finally, the CAM multiplies these weights with the input features channelwise to obtain the weighted feature output, thereby enhancing the model’s focus on important channel features.

The set of neighboring points of each point can be represented as

$$M_{p_{i}}=KNN(p_{i})$$ (1)

here, *KNN*(*p*_*i*_) represents the use of the KNN algorithm to find the K nearest neighbors of point ${𝒫}_{i}$. Specifically, the KNN algorithm calculates the distances between point ${𝒫}_{i}$ and other points, selects the K points closest to ${𝒫}_{i}$, and forms the neighborhood set $M_{p_{i}}$ of point ${𝒫}_{i}$.

$$f\left(p_{i}\right)=\max\text{pool}\left(\Gamma \left(M_{p_{i}}\right)\right)$$ (2)

here, Γ(•) represents the convolution operation, and $f\left(p_{i}\right)$ is the local neighborhood feature of ${𝒫}_{i}$ after convolution.

For each point ${𝒫}_{i}$ and its neighboring point ${𝒫}_{j}$, the feature difference $\Delta f_{ij}$ can be represented as

$$\Delta f_{ij}=f\left(p_{i}\right)-f\left(p_{j}\right)$$ (3)

Extraction of the difference information via the 2D convolutional layer: Using a 2D convolution kernel with a size of 1, the feature $f_{ij}^{\;\prime }$ after the convolution operation can be represented as

$$f_{ij}^{\;\prime }=Conv2D(1,\Delta f_{ij})$$ (4)

Feature max pooling: For each point *x*_*i*_ and its neighborhood $M_{x_{i}}$,the maximum value of the features in the neighborhood can be represented as

$$f_{\max}^{\;i}=\max_{j\in M_{p_{i}}}\left(f_{ij}^{\;\prime }\right)$$ (5)

Further extraction features via the 1D convolutional layer: The 2D convolution kernel is also set to 1, and the features obtained through the 1D convolutional layer are represented as

$$f_{1D}^{\;i}=Conv1D(1,f_{\max}^{\;i})$$ (6)

The adaptive average pooling feature $f_{avg}^{\;c}$ is represented as

$$f_{a\nu g}^{c}=\frac{1}{H\times W}\sum_{h=1}^{H}\sum_{W=1}^{w}f_{1D}^{i}$$ (7)

where H and W are the height and width of the feature map, respectively.

Channel compression and activation are obtained via

fcompressedc=ReLU(Conν1D(K2D,favgc)) (8)

here, ReLU denotes the Rectified Linear Unit function, defined as ReLU(x)=max(0,x), max(0,x) represents the larger value between x and zero.

Channel restoration and weight generation are obtained via

$$w^{c}=sigmoid\left(Conv1D\left(K_{3D},f_{compressed}^{\;c}\right)\right)$$ (9)

The weighted feature output is obtained via

$$f_{output}^{\;i}=f_{1D}^{\;i}⊙w^{c}$$ (10)

where ⊙ represents the channelwise multiplication operation.

### Main feature selection

After the feature output processed by the CAM is obtained, elementwise summation is performed for each feature branch, and weight scores are calculated via a multilayer perceptron (MLP) with nonshared weights. Let the feature branches be $F_{1},F_{2},...,F_{k}$ and their weight scores can be represented as

$$S_{i}=MLP(F_{i})$$ (11)

To further process these weight scores, the Softmax activation function is used to convert them into a probability distribution, represented as

$$w^{\prime }=softmax(\{S_{1},S_{2},...,S_{k}\})$$ (12)

Softmax is applied to normalize the weights, ensuring that the sum of all feature weights equals 1, facilitating subsequent weighting operations. This normalization not only highlights significant features but also simplifies loss computation, enabling the model to focus more on features that are important for the registration task.

**Fig 3:**
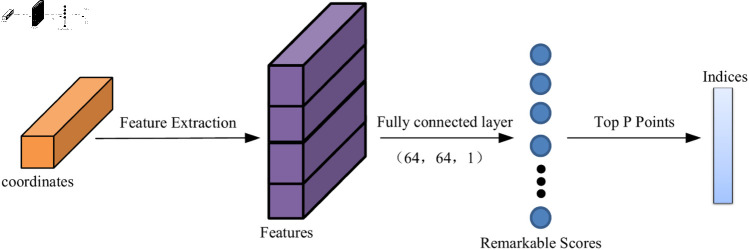
Main feature selection module.

Finally, the top P data points with the remarkable scores are retained, and the remaining points are removed [24]. In our network, we set P to be one-sixth of the total number of points in either the source or target point cloud. The sampled source point cloud is denoted $X^{\prime }=\left\{x_{i}^{\prime }\in R^{3}|i=1,2,\cdots P\right\}$,and the target point cloud is denoted $Y^{\prime }=\left\{y_{j}^{\prime }\in R^{3}|j=1,2,\cdots P\right\}$. The main feature selection module is shown in Fig 3.

### Feature fusion

In point cloud registration tasks, relying solely on local features is often associated with significant drawbacks. Local features can capture details of each small region in the point cloud but often fail to provide global context information. This means that the model may misunderstand the overall shape and structure, leading to insufficient judgment of complex shapes. Additionally, local features are susceptible to noise and occlusion, which can lead to instability in feature extraction and affect the accuracy of matching. To enhance feature discriminability and capture both local and global contextual information, a feature fusion module is incorporated. The features from both the source and target point clouds are concatenated to form a multiscale feature matrix. This combined feature vector not only captures the local characteristics of the point clouds but also includes distance information, which is obtained by computing the distance differences between the source and target point clouds [2]. The feature fusion matrix is

$$G^{\left(n\right)}(i,j)=(P_{X}(i)⊕P_{Y}(j)⊕\|x_{i}^{\prime }-y_{j}^{\prime }\|⊕\frac{x_{i}^{\prime }-y_{j}^{\prime }}{\left\|x_{i}^{\prime }-y_{j}^{\prime }\right\|})$$ (13)

where *P*_*X*_(*i*) is the geometric feature of $x_{i}^{\prime }\in X^{\prime }$; *P*_*Y*_(*j*) is the geometric feature of $y_{i}^{\prime }\in Y^{\prime }$; ⊕ denotes the concatenation operation; and n represents the number of internal iterations of multiscale features after the distance features are updated.

### Generation of matching probabilities

#### Similarity score analysis.

The similarity is calculated by constructing the multiscale feature matrix mentioned in the feature fusion chapter. This process is crucial for the effectiveness of point cloud registration. The features of the source and target point clouds are processed through an embedding network to generate high-dimensional feature representations. These features are then stacked spatially to form a multiscale feature matrix that integrates the embedded features of the source and target point clouds.

The Euclidean distance between the source and target point clouds is calculated to construct the multiscale feature matrix. These distance features not only reflect the spatial relationship between the two but also provide additional information for similarity evaluation. By combining the embedded features and distance features, the model can better understand the similarity between point clouds during the matching process.

The fused features undergo a sequence of 1×1 2D convolution operations, resulting in a single-channel image with the same spatial dimensions at the output. This process is analogous to applying a multilayer perceptron to each position of the fused feature vector. The similarity matrix, denoted as *T*^(*n*)^(*i*,*j*),is obtained by applying the Softmax function to each row of the single-channel image. Each row of *T*^(*n*)^(*i*,*j*) defines a normalized probability distribution, which represents the probability of *y*_*i*_ corresponding to *x*_*i*_.

To find the corresponding pairs, an argmax operation is applied to each row of the similarity matrix, yielding a set of corresponding pairs $\left\{(x_{i}^{\prime },\dot{x})|\forall x_{i}^{\prime }\in X\right\}$,where $\dot{x}$ is the point in the target point cloud with the highest probability corresponding to $x^{\prime }$.

#### Effectiveness score analysis.

**Fig 4:**
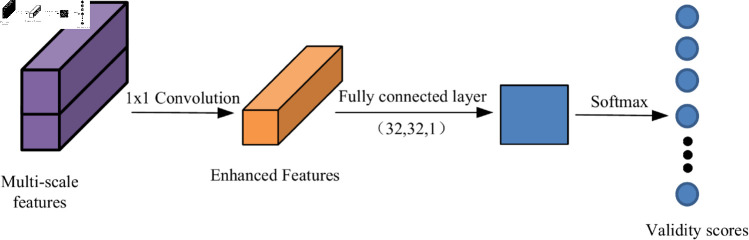
Process of the effectiveness scoring network.

Selecting feature-significant data points from the source and target point clouds can greatly improve the registration efficiency. However, this may also lead to the omission of correct correspondences for some points in the source point cloud in the target point cloud [24]. To address this issue, an effectiveness scoring network is proposed to assess the reliability of the matching results. The network analyzes the point pair relationships in the similarity matrix and calculates the effectiveness score for each corresponding point, thereby optimizing the matching process. The workflow of the effectiveness scoring network is shown in Fig 4.

The network performs a convolution on the input multiscale features, using 1×1 convolution layers to extract features and obtain enhanced feature representations. The convolution results after max pooling are processed by a multilayer perceptron (MLP) to compute the effectiveness score for each point.

$$c_{i}=\partial (MLP(\max(G^{\left(n\right)}(i,j)))$$ (14)

where ∂(•) is the sigmoid function.

The importance weight *d*_*i*_ for each point is calculated on the basis of the effectiveness score as follows:

$$d_{i}=\frac{c_{i}\left\langle c_{i}\geq median(c)\right\rangle }{\sum_{i}c_{i}\left\langle c_{i}\geq median(c)\right\rangle }$$ (15)

The weighting process uses the indicator function ⟨•⟩. The weight represents the importance of individual points in the point cloud, with key points being identified based on their effectiveness scores. Points with low validity scores are assigned a weight of zero. The remaining points are weighted on the basis of their validity scores.

The weights are combined, and singular value decomposition (SVD) [25] is used to compute the rotation matrix and translation vector of the point cloud. The registration results are optimized. The formula is given as follows:

$$R(n),t(n)=\arg\min_{R,t}\sum_{i}d_{i}∥{Rx}_{i}^{\prime }+t-{\dot{x}}_{i}{∥}^{2}$$ (16)

### Loss function

Four loss functions are defined in total. The initial loss function is the supervision loss $\gamma_{sl}$ for the feature extraction module. It consists of two main components, rigid transformation loss and feature consistency loss, which are defined as follows.

$$\gamma_{sl}=\left(\frac{1}{N}\sum_{i=1}^{N}\|Rx_{i}+t-y_{j}{\|}_{2}^{2}\right)+\left(\frac{1}{N}\sum_{i=1}^{N}\|f_{output}^{\;i}\left(x_{i}\right)-f_{output}^{\;j}\left(y_{j}\right){\|}_{2}^{2}\right)$$ (17)

here, $f_{output}^{\;i}(x_{i})$ and $f_{output}^{\;j}(y_{j})$ represent the weighted features of the source and target point clouds output by the feature extraction and enhancement module.

The supervised loss function for the main feature selection is defined as follows:

$$\gamma_{ml}=\frac{1}{M}\sum_{i=1}^{M}{\left\{g(i)-\sum_{j=1}^{M}G^{\left(n\right)}(i,j)log\left(G^{\left(n\right)}(i,j)\right)\right\}}^{2}$$ (18)

here, g(i) represents the validity score of the i-th point in the source point cloud set.

For the feature fusion process, the supervised loss for feature registration, which combines distance features to assess the relationship between two points, is defined as

$$\gamma_{fl}=-\frac{1}{ℳ}\sum_{i=1}^{M}\log\left(G^{\left(n\right)}\left(i,j^{•}\right)\times h\right)$$ (19)

where $j^{•}$ is the point in the target point cloud that is closest to the true value of point $x_{i}^{\prime }$ in the source point cloud. $h=\left\langle {\left\|R^{*}x_{i}^{\prime }+t^{*}-y_{j}^{\prime }\right\|}^{2}\leq r^{2}\right\rangle$, where h is an indicator function used to determine whether the correspondence between these two points in the source and target point clouds meets the condition.

The registration validity supervision loss function is defined as

$$\gamma_{rl}=-\frac{1}{M}\sum_{i=1}^{M}\left(\hat{h}\cdot \log(c_{i})+(1-\hat{h})log(1-c_{i})\right)$$ (20)

where $\hat{h}=\left\langle \left\|R^{*}x_{i}^{\prime }+t^{*}-y_{argmax_{j}G^{\left(n\right)}(i,j)}\right\|\leq r^{2}\right\rangle$ and where $\hat{h}$ is an indicator function used to determine whether the distance between two points satisfies the correspondence requirement given the true value transformation.

The total loss function $\gamma_{tl}$ is defined as follows:

$$\gamma_{tl}=\lambda_{1}\gamma_{sl}+\lambda_{2}\gamma_{ml}+\gamma_{fl}+\gamma_{rl}$$ (21)

To simplify hyperparameter management, two weight parameters, $\lambda_{1}$ and $\lambda_{2}$,are introduced. The weight parameter $\lambda_{1}$ adjusts the contribution of the feature enhancement loss, whereas $\lambda_{2}$ is the weight for the main feature selection loss. Since local geometric features are more important than distance features during the early stages of registration,$\lambda_{2}$ is set to 1 in the first iteration and then set to 0 in subsequent iterations.

## Experimental analysis

### Configuration of the experimental environment

The Python processing language was used for the experiments in this work, and the environment was configured as the PyTorch deep learning framework. The weight decay parameters were set to 0.001 during the model training process. The Adam optimizer was selected, with an initial learning rate of 0.0001. A learning rate scheduler was applied throughout the training, reducing the rate to 10% of the original by the 30th epoch. The value of M was set to 0.6.

We evaluated the proposed algorithm on the ModelNet40 dataset and the point cloud dataset publicly released by Stanford University, which is based on real-world object scans. The ModelNet 40 dataset comprises 12,311 gridded CAD models distributed across 40 categories. The training set includes 9,843 models, while the test set contains 2,468. For each object, 1,024 points were randomly sampled to form the point cloud. Additionally, random rotations within the range [0, 45] degrees and translations in the range [-0.5, 0.5] were applied to each point cloud. The original point cloud is designated as the source, and the transformed version is referred to as the target. To create partially overlapping point clouds, we fixed a random point where two point clouds were far apart and kept the 768 nearest points from that fixed location for each cloud. All angle measurements in our experiments are given in degrees.

### Results and analysis

The methods in this paper are compared with traditional algorithms (e.g., iterative nearest-point ICP methods [6], FGR methods [11], and GO-ICP methods [26]) and deep learning-based methods (e.g., PointNetLK [13], DCP [16], PRNet [21], and IDAM [2]). All deep learning-based methods are trained using the same training set, with the evaluation metrics being the root mean square error (RMSE) and mean absolute error (MAE) for both the rotation matrix and translation vectors. For the rotation matrix, the rotation RMSE (RMSE(R)) and rotation MAE (MAE(R)) are measured in degrees. Additionally, the translation vectors are assessed using the translation RMSE (RMSE(t)) and translation MAE (MAE(t)).

To verify the validity of the methods in this paper, we designed and implemented three different experimental scenarios. These experiments aim to systematically evaluate the model’s registration accuracy and generalization ability in invisible shapes, invisible categories, and noisy environments.

#### Evaluation of point cloud registration for invisible shapes.

**Fig 5:**
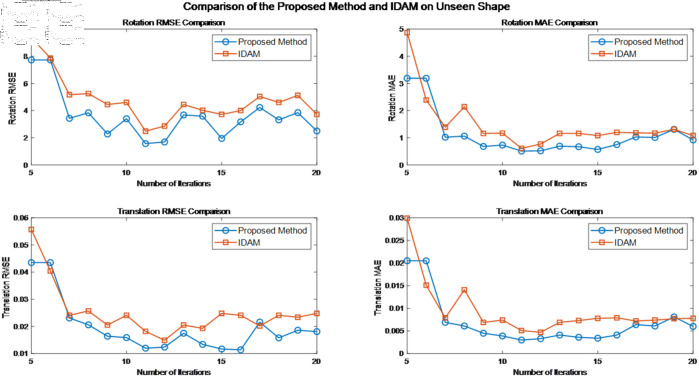
Comparison of registration performances on unseen shapes.

The model was trained using the ModelNet40 dataset and tested on its corresponding test set. Both the training and test sets include point clouds from all 40 categories. The experiments were conducted to assess the model’s ability to generalize to unseen point clouds. The results can be found in Table 1. Furthermore, Fig 5 compares the registration performance of the IDAM algorithm with that of the proposed method on previously unseen shapes.

**Table 1:** ModelNet40: Registration results for an invisible shape point cloud.

Model	RMSE (R)	MAE (R)	RMSE (t)	MAE (t)
ICP	33.12	26.21	0.289	0.234
PointNetLK	17.32	7.67	0.058	0.047
Go-ICP	13.84	3.58	0.032	0.013
FGR	11.32	2.78	0.027	0.006
DCP	7.38	5.42	0.041	0.032
PRNet	3.52	1.76	0.019	0.012
IDAM	2.49	0.61	0.018	0.005
Ours	1.57	0.51	0.012	0.002

Traditional algorithms, e.g., ICP, have relatively poor registration results and high errors in the absence of an accurate initial position; although Go-ICP introduces branch and bound methods to alleviate the local minimum problem, it is still sensitive to the initial position. Despite achieving a high level of accuracy, fast global registration (FGR), which uses geometric features for registration, still has some errors compared with deep learning-based methods.

Deep learning methods, e.g., DCP and PRNet, use geometric features for registration with improved performance. IDAM further improves the registration result by mixing features, but the feature information extracted by it is relatively limited. In comparison, the approach introduced in this paper demonstrates superior ability to capture the local geometric features of the point cloud, achieving higher registration accuracy and the lowest RMSE and MAE values among all the methods tested.

#### Evaluation of point cloud registration for invisible categories.

**Fig 6:**
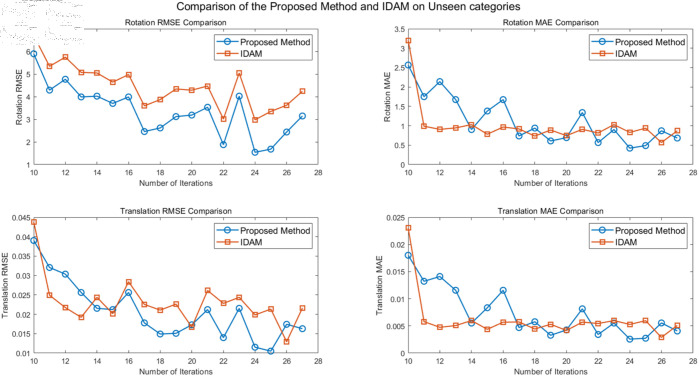
Comparison of registration performances on unseen categories.

Generalization experiments were conducted for point cloud registration on unseen categories, where the first 20 categories were used for training and the other 20 for testing. The registration results of different algorithms on point clouds from the unseen categories of the ModelNet40 dataset are shown in Table 2. Additionally, Fig 6 provides a comparison between the proposed algorithm and IDAM.

**Table 2:** ModelNet40: Registration results for the invisible point cloud categories.

Model	RMSE (R)	MAE (R)	RMSE (t)	MAE (t)
ICP	33.82	26.46	0.291	0.238
PointNetLK	21.75	8.97	0.061	0.033
Go-ICP	12.79	2.97	0.034	0.011
FGR	11.32	2.78	0.027	0.006
DCP	9.98	1.99	0.040	0.008
PRNet	4.85	2.27	0.017	0.011
IDAM	2.97	0.82	0.019	0.005
Ours	1.54	0.42	0.011	0.002

#### Evaluation of point cloud registration for invisible shapes with Gaussian noise.

**Fig 7:**
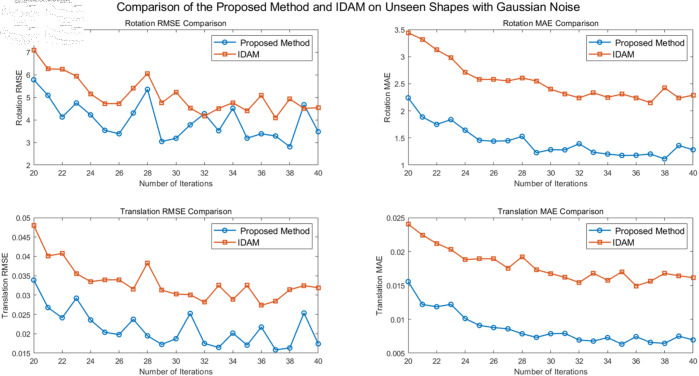
Comparison of registration performances on unseen shapes with gaussian noise.

Registration tests were performed on point clouds of invisible shapes with Gaussian noise included. This is because scanned models in real scenes often contain noise. In this work, random Gaussian noise with a standard deviation of 0.01 was added to all shapes, and the random noise was cropped to [-0.05, 0.05]. The samples from each category were split into training and testing sets in equal proportions, with 50% of the samples allocated to the training set and the other 50% to the testing set. This strategy ensures the balance between the training set and the test set, enhances the evaluation ability of the model to new categories, and further improves the generalization ability of the model. In Table 3, a comparison of various methods is conducted on the ModelNet40 dataset with Gaussian noise added to unseen shapes. In Fig 7, the proposed algorithm is compared with the well-performing IDAM algorithm.

**Table 3:** ModelNet40: Registration results for an invisible point cloud with gaussian noise.

Model	RMSE (R)	MAE (R)	RMSE (t)	MAE (t)
ICP	36.67	27.11	0.298	0.257
PointNetLK	20.79	9.72	0.069	0.038
Go-ICP	13.46	3.13	0.032	0.041
FGR	28.21	13.99	0.071	0.039
DCP	9.29	7.14	0.079	0.063
PRNet	5.38	2.97	0.035	0.019
IDAM	4.16	2.23	0.028	0.015
Ours	2.81	1.11	0.016	0.006

To further analyze the robustness of our method under different noise levels, we conducted additional tests on registration performance with standard deviations of 0.02 and 0.03. The experimental results are shown in Table 4.

**Table 4:** ModelNet40: Comparison of registration errors at different noise levels.

Standard deviation	RMSE (R)	MAE (R)	RMSE (t)	MAE (t)
0.01	2.81	1.11	0.016	0.006
0.02	3.47	1.53	0.022	0.010
0.03	4.09	1.95	0.031	0.014

Based on the experimental findings,at a standard deviation of 0.01, the approach proposed in this paper outperforms both traditional and deep learning algorithms in point cloud registration under Gaussian noise conditions. Compared with traditional algorithms, e.g., ICP and FGR, the method in this work significantly reduces both the rotation error and translation error, demonstrating stronger robustness to noise. Moreover, compared with deep learning algorithms, e.g., DCP, PRNet and IDAM, the method in this paper can better filter the noise points and nonoverlapping points to maintain high registration accuracy in noisy environments. However, in practical applications, the noise level of scanned data may vary. To address this, we further tested the registration performance at standard deviations of 0.02 and 0.03. The experimental results show that as the noise level increases, the rotation and translation errors also rise. This indicates that noise has a certain impact on registration accuracy, but the overall increase in error remains controllable, demonstrating that our method maintains good robustness under noise interference.

#### Computational efficiency.

This study evaluates the computational efficiency of different point cloud registration algorithms on the ModelNet40 test set, focusing on comparing the average processing time of several representative algorithms for a single-frame point cloud. The evaluation covers three typical point cloud sizes: 512, 1024, and 2048 points, as shown in Table 5 (unit: seconds). The results highlight that deep learning methods exhibit a substantial advantage in computational efficiency over traditional algorithms. Further analysis indicates that deep learning models relying on feature extraction often incorporate redundant nonoverlapping points and weakly discriminative feature points during geometric feature representation, which can considerably escalate the computational complexity of the registration process. The dual-layer feature selection mechanism introduced in this study effectively enhances computational efficiency while preserving registration accuracy.

**Table 5:** Running time of different registration algorithms.

Sampling number	ICP	DCP	IDAM	Ours
512	0.023	0.005	0.019	0.015
1024	0.043	0.009	0.024	0.019
2048	0.086	0.016	0.041	0.027

#### Point cloud registration experiment based on the Stanford dataset.

To further validate the generalization ability of the proposed algorithm, we conducted experiments on the Stanford 3D scanned point cloud dataset released by Stanford University. Compared to ModelNet40, this dataset features more uneven point cloud distributions, making it more challenging. For this experiment, we selected the Bunny model from the Stanford dataset and downsampled it to 2048 points. To generate the target point cloud, we applied random rotations within the range of [0°, 45°] along each coordinate axis and random translations within the range of [–0.05, 0.05] to each point cloud. The proposed algorithm was compared against two state-of-the-art methods, DCP and IDAM, with the detailed results presented in Table 6.

**Table 6:** Registration performance on the Stanford dataset.

Model	RMSE (R)	MAE (R)	RMSE (t)	MAE (t)
DCP	8.44	6.41	0.018	0.015
IDAM	3.23	2.32	0.012	0.006
Ours	2.34	1.78	0.008	0.003

#### Point cloud registration experiments with Gaussian noise on the Stanford dataset.

We added random Gaussian noise with a standard deviation of 0.01 to the real data model, constrained within the range of [-0.05, 0.05], consistent with the experimental setup for " Evaluation of point cloud registration for invisible shapes with Gaussian noise." The results of adding Gaussian noise to the Stanford dataset are shown in Table 7.

**Table 7:** Registration performance with noise on the Stanford dataset.

Model	RMSE (R)	MAE (R)	RMSE (t)	MAE (t)
DCP	11.31	8.26	0.031	0.025
IDAM	5.80	4.18	0.023	0.016
Ours	3.65	2.63	0.017	0.011

#### Visualization.

**Fig 8:**
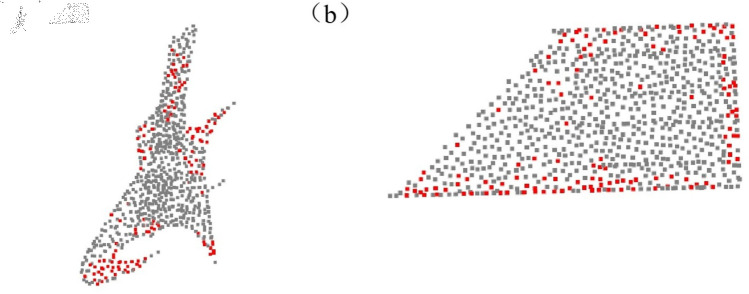
Visualization of key feature points. (a) Visualization of key feature points for object 1. (b) Visualization of key feature points for object 2.

**Fig 9:**
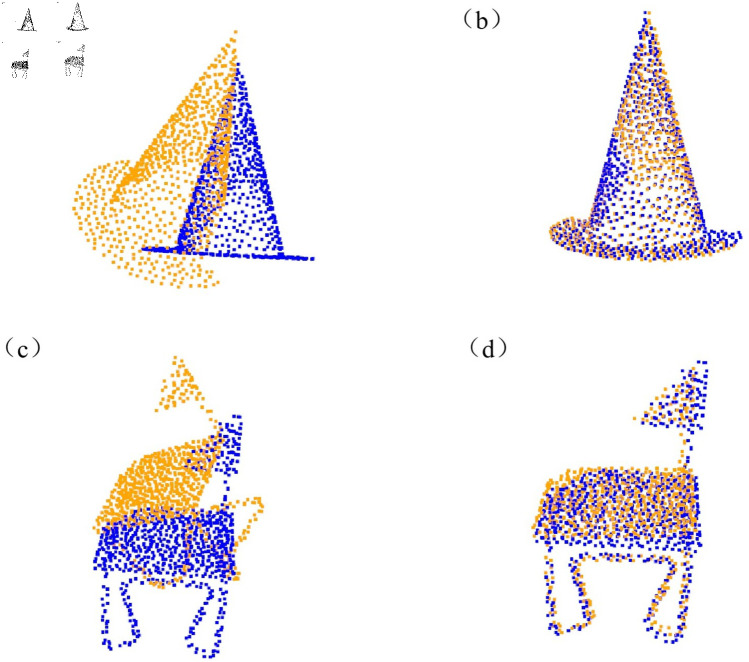
Registration results on the ModelNet40 dataset. (a) Initial point cloud position of object A. (b) Registered point cloud result of object A. (c) Initial point cloud position of bject B. (d) Registered point cloud result of object B.

**Fig 10:**
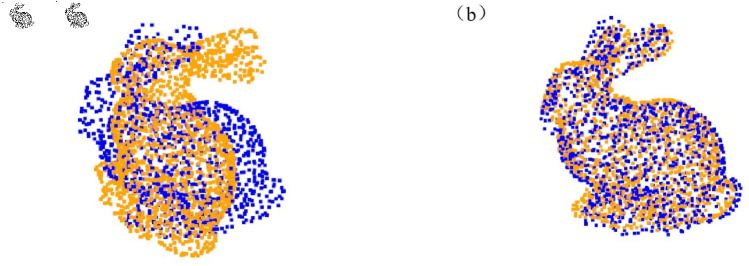
Registration results on the Stanford dataset. (a) Initial point cloud position. (b) Registered point cloud result.

In the visualization section, we first present the effect of the feature selection module. As shown in Fig 8, we visualized the point cloud, retaining the top 128 points with the highest importance scores, and highlighted these key points in the visualization. Next, we present the registration results of the algorithm on the ModelNet40 dataset and the Stanford point cloud dataset, as shown in Fig 9 and Fig 10.

### Fusion research

A fusion analysis was conducted to evaluate the contribution of each module in the proposed point cloud registration method. By gradually removing or replacing different modules and observing their impact on the overall registration performance, we conducted experiments in the ‘invisible shapes with Gaussian noise’ setting as described in the ‘Results and Analysis’ section.

In this experiment, for the fusion of the first part, we replaced the feature extraction and enhancement part with the traditional feature extraction method (FPFH). In the absence of a primary feature selection process, random point selection was used instead. The feature extraction and enhancement part is denoted FE, the main feature selection part is denoted MF, the feature fusion part is denoted FF, and the match probability generation part is denoted MP. The FPFH method is denoted BS. Table 8 presents the results of different combinations of key components on the ModelNet40 dataset.

**Table 8:** Results of different combinations of key components on ModelNet40.

Model	RMSE (R)	MAE (R)	RMSE (t)	MAE (t)
BS+MF+FF+MP	4.47	1.78	0.027	0.011
FE+FF	5.52	1.97	0.032	0.013
FE+MF +FF	4.50	1.84	0.025	0.011
FE+MF+FF+MP	2.81	1.11	0.016	0.006

The experimental results show that the method proposed in this work significantly improves both the RMSE and the MAE metrics, which proves the enhancement of feature extraction in overlapping regions.

## Discussion

### Algorithm performance and advantages

The point cloud registration algorithm proposed in this paper, based on KNN and CAM, demonstrates significant advantages in terms of accuracy and robustness compared to traditional point cloud registration methods like ICP, DCP, and IDAM. In handling overlapping regions, traditional methods often face significant errors due to insufficient local features. In contrast, our method, by combining the KNN algorithm and channel attention mechanism, can more accurately capture local geometric structures, thereby reducing mismatching situations.

The KNN algorithm effectively enhances the representation of local structures through neighborhood information, while the channel attention mechanism adaptively assigns higher weights to important regions during the feature fusion process. This weighting mechanism allows for more precise attention to local details during the registration process, significantly improving registration accuracy. In multiple complex scenarios and under noise influence, the performance of this method is also more robust compared to traditional methods. For example, on the more complex point cloud dataset ModelNet40, experimental results show that, even with significant point cloud overlap and noise, this method is still able to maintain low registration errors.

### Innovation in feature extraction and enhancement

Traditional point cloud registration methods often rely on manually designed features, such as FPFH, which provide some level of local geometric information but tend to overlook global context and local transformations in complex scenes. In contrast, the feature extraction method proposed in this paper, based on KNN and channel attention mechanisms, is able to better capture the complex local geometric features of point clouds.

he KNN algorithm, by searching the neighborhood of each point, not only preserves the information of local structures but also enhances the sensitivity to geometric details. CAM, on the other hand, highlights important geometric information by weighting the features, allowing the registration process to more accurately locate key points. Particularly in overlapping regions, the combination of local details and global context significantly improves registration accuracy, compensating for the limitations of traditional methods that rely solely on local geometric information.

Additionally, the dynamic weighting strategy in the feature extraction process enhances the flexibility and adaptability of the algorithm, making its performance more stable across different scenarios. This approach, combining local and global features, demonstrates innovation in the field of feature extraction and can serve as a reference for subsequent point cloud processing tasks.

### Limitations of the model and directions for improvement

Although the proposed method demonstrates clear advantages in point cloud registration, it also has certain limitations. First, due to the introduction of KNN and convolution operations, the method is computationally complex. Particularly when handling large-scale point cloud data, the computational overhead can become a bottleneck. Therefore, how to balance computational efficiency and accuracy remains an issue that needs to be addressed.

Secondly, although the channel attention mechanism effectively improves the accuracy of feature selection, there is still a risk that some key features may be misclassified as noise in complex scenarios. To address this issue, future work could incorporate more self-supervised learning or reinforcement learning strategies to further optimize the accuracy of feature selection.

### Experimental analysis and discussion

Through experiments on the ModelNet40 dataset, the proposed method significantly outperforms traditional ICP and DCP methods in terms of point cloud registration accuracy and robustness. Especially when dealing with point cloud data that has higher noise and greater overlap, our method demonstrates lower RMSE and MAE, indicating its ability to effectively reduce registration errors.

Compared to the IDAM method, although IDAM can handle overlapping regions and noise, it has certain bottlenecks in computational efficiency and may not accurately capture local features in some complex scenarios.

The algorithm proposed in this paper, based on KNN and channel attention mechanisms, effectively avoids this issue through more precise feature extraction and weighting mechanisms.

However, despite this, the method still has certain limitations when handling extremely complex scenarios. For example, when dealing with large amounts of point cloud data, the demand for computational resources remains high. Therefore, how to improve computational efficiency while ensuring accuracy will be an important direction for future research.

### Potential applications

The point cloud registration method proposed in this paper has broad application prospects, particularly in fields such as autonomous driving, robot navigation, and 3D reconstruction. In autonomous driving, point cloud registration technology is one of the key components for precise map construction and obstacle detection. By improving the accuracy of point cloud registration, the proposed method can better support real-time environmental perception and decision-making.

In robot navigation, point cloud registration helps the robot accurately perform path planning and obstacle avoidance. Especially in complex indoor and outdoor environments, the proposed method can effectively address challenges such as point cloud overlap and noise, thereby improving navigation accuracy. Additionally, the application of the proposed method in 3D reconstruction also holds potential, especially in multi-view reconstruction, where precise point cloud registration can effectively reduce errors and improve reconstruction accuracy.

## Conclusion

The overlapping point cloud registration algorithm based on KNN and the channel attention mechanism presented in this work effectively improves the accuracy and robustness of point cloud registration. First, the local features of the point cloud in different domains are searched by KNN to enhance the ability to describe the local geometrical structure and identify the key points with rich features, thus improving the registration efficiency. The CAM module dynamically adjusts the importance of features on this basis, enabling the network to flexibly capture the relationships between key points to further enhance feature representation. Second, by combining the local geometric and distance features of the point cloud and utilizing the interaction of different point pairs of features, the impact of the initial position on the alignment performance is reduced; thus, the registration accuracy is improved. Finally, to balance performance and efficiency, the validity scoring network was designed to reduce the impact of incorrectly removing counterparts during critical point sampling on registration performance. Although our method has achieved promising results in point cloud registration accuracy, the partial overlap of point cloud data remains a challenge in the registration process. The accumulation of registration errors is often related to mismatches in non-overlapping regions. Therefore, accurately estimating the overlapping regions and incorporating them into the registration process can provide more constrained global information, reducing the propagation of local errors. Future research could explore the application of overlap region estimation in point cloud registration. designing new optimization strategies to integrate overlap information into the registration process, thereby improving the accuracy of initial pose estimation. Although this direction holds potential, its practical application still faces several challenges. For instance, accurate estimation of overlap regions may be affected by uneven point cloud density, noise, or occlusions. Additionally, in automated point cloud processing scenarios, overlap estimation could reduce reliance on manual intervention, enhance matching efficiency, and provide more stable registration results for large-scale point cloud data processing.
