# Correction: Overlapping point cloud registration algorithm based on KNN and the channel attention mechanism

**DOI:** 10.1371/journal.pone.0333601

**Published:** 2025-09-30

**Authors:** Yangzhuo Chen, Fengjiao Guo, Jingang Liu, Siling Dai, Jia Huang, Xiaowen Cai

There are errors in the author affiliations. The correct affiliations are as follows:

Yangzhuo Chen^1,2,3^, Fengjiao Guo^1^, Jingang Liu^2^, Siling Dai^1^, Jia Huang^4^, Xiaowen Cai^1^

**1.** School of Automation and Electronic Information, Xiangtan University, Xiangtan, China, **2.** School of Mechanical Engineering and Mechanics, Xiangtan University, Xiangtan, China, **3.** Hunan Shaofeng Institute of Applied Mathematics, Xiangtan, China, **4.** Xingxiang College of Xiangtan University, Xiangtan, China
